# Supplementary figures and images for: Combining predictive models with future change scenarios can produce credible forecasts of COVID-19 futures
Source: PLoS One. 2022 Nov 15;17(11):e0277521. doi: 10.1371/journal.pone.0277521 (PMC9665358; doi:10.1371/journal.pone.0277521)

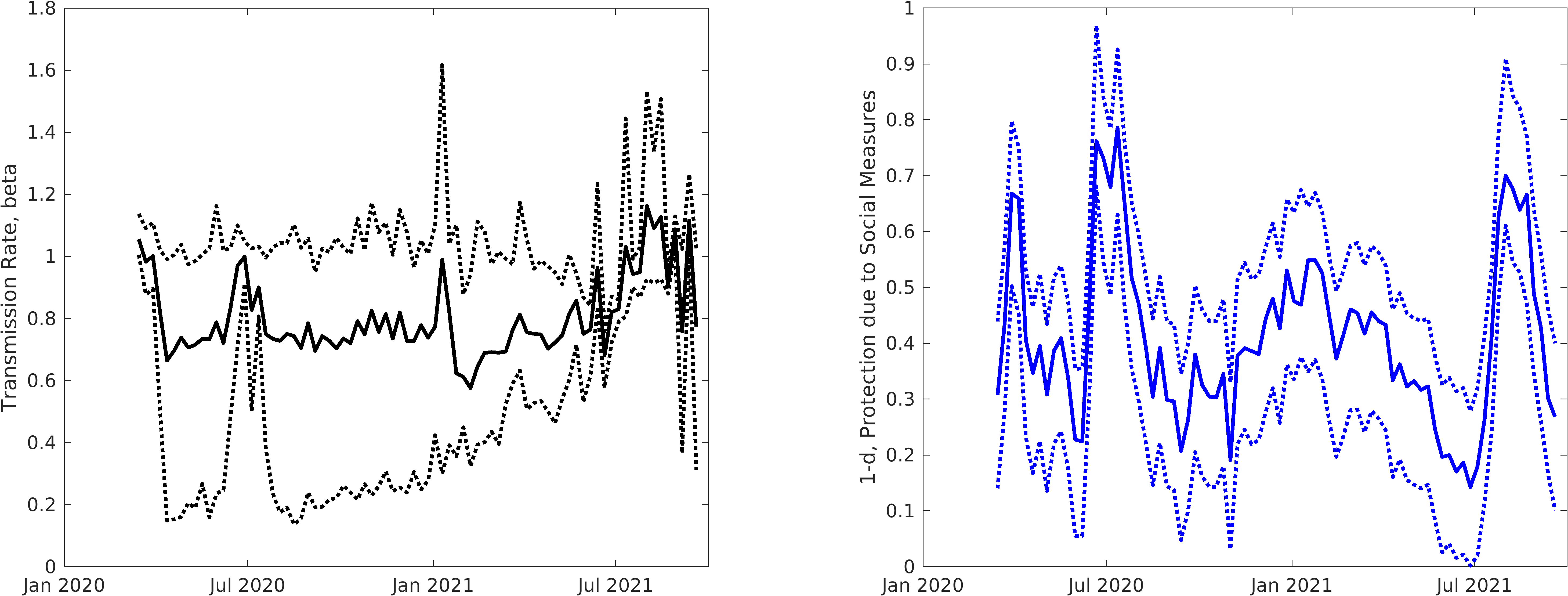

Supplement: S1 Fig — The transmission rate is an averaged rate over alpha, delta, and all other variants. The priors on the d parameter are informed by Google Trends search data, as described in the main text. (TIFF) [file pone.0277521.s001.tiff]

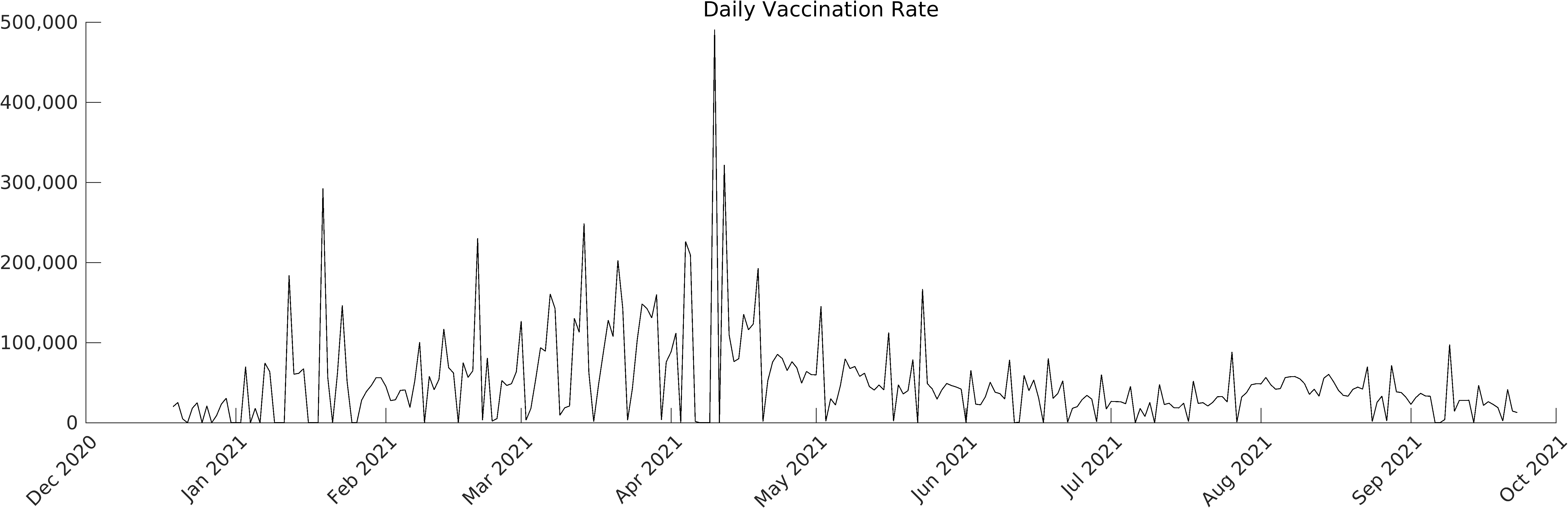

Supplement: S2 Fig — (TIFF) [file pone.0277521.s002.tiff]

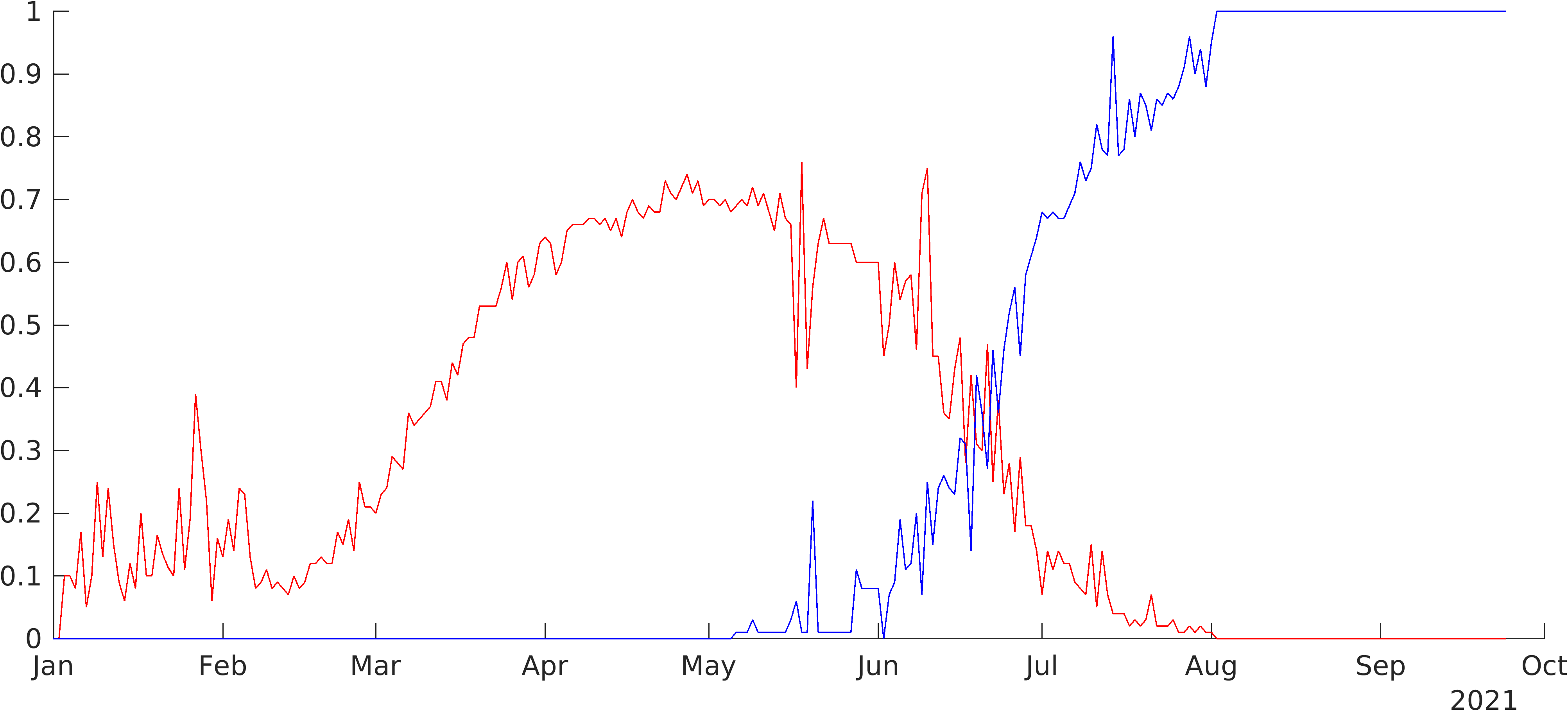

Supplement: S3 Fig — (TIFF) [file pone.0277521.s003.tiff]

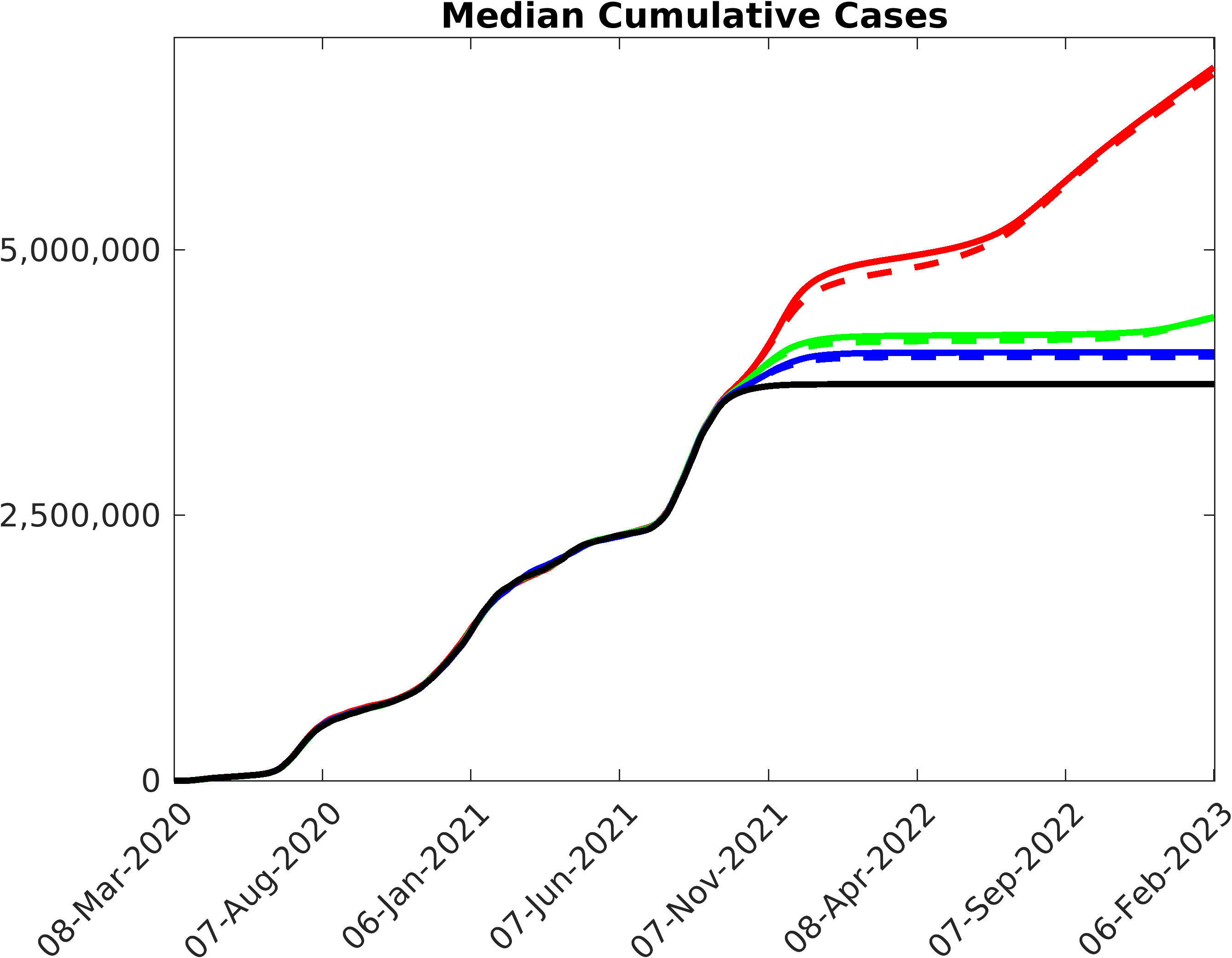

Supplement: S4 Fig — The median model prediction given estimates of social measures and vaccination rate as of Sept. 24th, 2021 is given in black, while 1 yr, 2.5yr, and 5yr immunity waning periods are shown in red, green, and blue, respectively. The solid lines represent estimated vaccination rate, while the dashed lines represent a 1.5x increase in vaccination rate. (TIFF) [file pone.0277521.s004.tiff]
