# Supplementary material for: Combining predictive models with future change scenarios can produce credible forecasts of COVID-19 futures
Source: PLoS One. 2022 Nov 15;17(11):e0277521. doi: 10.1371/journal.pone.0277521 (PMC9665358; doi:10.1371/journal.pone.0277521)
Supplement: Attachment — Submitted filename: Review.pdf [file pone.0277521.s005.pdf]

The manuscript ”Combining predictive models with future change scenarios can produce credible forecasts of COVID-19 futures” by Newcomb analyses SARS-CoV-2 pandemic in the State of Florida. The authors develop an extended SEIR compartmental model that they calibrate to data, and then use to investigate future scenarios. Such predictions are important to anticipate the pandemic evolution, in order to timely change and adapt social measures. The authors study the impact of vaccinations, the consequences of immunity waning, and the impact of possible new variants. They conclude that new variants and loss of immunity may induce repeated waves of infection, but the severity of these waves is probably small due to a high levels of immunity acquired by vaccinations and natural infections.

In general, the study is interesting and well written. However, I have some comments that the authors might want to address in order improve the comprehension of their work:

- As I understand, there is no age stratification, and only one parameter for the social interaction level. At least in the discussion the authors should comment on this, and how this affects their conclusions, since social interactions, infections dynamics and severity of an infection strongly depends on age.

- The authors should explain in more detail how the sequential fitting is done. Which parameters are kept fix, which are fitted, and what is the rationale behind their procedure. For example, sequential fitting procedure has been implemented for France in order to control the pandemic with social measures (https://www.medrxiv.org/content/10.1101/2021.07.28.21260870v2). After fixing infection parameters, only parameters for social interactions are changed for the sequential fitting (until vaccinations and new variants emerge). However, since in this model there seems only one parameter for social interactions, is this the parameter that has is mostly changed ? How does their method compare to the one implemented for France ?

- Why only daily confirmed case and mortality data is used for calibration, as stated in the sequential model calibration section ? Why not also the daily number of hospitalisations shown in Figure 4, for example ? How it comes that the model reproduces the hospitalisation data in Figure 4a (red circles), if this data has not been used for calibration ?

- Figure 2A: The authors should describe in the caption what are solid and dashed lines. Since panel (a) is about calibration, I suggest to stop the timeline at September 24 20221, since predictions are shown in (b).

- Figure 2b: The color coding should be as in (A), thus, the black solid line should be data.
  - The color coding is confusing, I do not see yellow and blue shadings.
  - There is no need to start the timeline already in Oct 2020, since it is the same in (a).
  - The y axis should be restricted as in (a), there is no information beyond that range. The peak of the blue curve can be given in the caption.
  - What is the purpose of adding the case number 22400 for August 26 to the panel ? Does it convey important information ? If yes, then it should be explained in the caption. If not, then it should be removed.
  - In the text, the figure is mentioned as Fig. S2b, which should be corrected

- Figure 3: I suggest to add Fig 3 to Fig 2 as panels (c) and (d), to gather all relevant information in one figure.

- Is there data than can be compared to the estimation of 30-35% for the immune population in March 2021 ?

- Figure 4: The figure is confusing, there are many solid and dashed curves, the yellow and blue bands look brown and violet.
  - Why all these peaks in the violet band ? Why is the violet band so large ?
  - Is there no hospitalisation and death data before June 2021 ?
  - What is the point of highlighting these numbers for August 30 September 8 in the two panels

- Figure 5: Are the simulations the same as in Fig 2, or are they refitted to the data for each waning period ?
  - How are the waning numbers calculated ? With an exponential distribution with time constants 1, 2.5 and 5 years ?

  - In Fig 2 without waning, the release of socials measures did not lead to a pandemic increase. Why such a strong reaction now with waning, what generates the instability after September 24 in presence of waning ? Why are the peaks occurring so shortly after September 24 although the waning periods are much larger ? The brief explanation given in the text is not sufficient for a comprehension.
  - It would be more comprehensive if the color coding in this figure would be as in Fig 5, thus, black and blue colors distinguish the two scenarios.

- I suggest to merge Fig 6 with Fig 5

- "We calculated and used the RMSE values of fits of our models to the case data observed over a 4-week period around the peak of the 4th (delta) wave to detect signals for the emergence and operation of waning immunity." To this reviewer it is unclear what exactly has been done.

- Figure 7: It is unclear how all the different curves are obtained. Which parameters are varied, and how ? Before September 2021, all the different scenario reproduce the data. After September 2021, they start to diverge. Does this mean, that the previous data was not sufficient to constrain all the model parameters ? Which parameters are most important for the divergence observed after September 2021 ? Which additional data would be helpful to constrain these parameters to reduce the prediction divergence?

- Figure 8: Same as Figure 7. Which parameter uncertainties or fluctuations causes the large divergence seen after September ?

- Section "Impact of new variants": I did not understand what exactly the parameter variance maintenance mechanism does, and how it affects the model.

- Figure 9a: Data is now available until February 2022. Why has the model not been calibrated to reproduce the whole data, since the simulation from September until January does not agree well with the data. Why ? In principle, the model should be calibrated to accurately reproduce all available data, before being applied and modified to make predictions. Such a procedure that continuously calibrates the model to

available data in order to make the best predictions has been developed for France (https://www.medrxiv.org/content/10.1101/2021.07.28.21260870v2).

- "Given that the best prediction was afforded by the November 1st emergence model, the data also suggests that a lead time of at least 6 weeks is required for the provision of such information to allow the reliable making of such a prediction." Is 6 weeks something general, or specific to this model ? What are the implications of this for reliable predictions ? We need past data from at least 6 weeks ?

- The Discussion is quite lengthy. I suggest to make it more crisp and to better highlight the main findings.
