# Supplementary material for: Combining predictive models with future change scenarios can produce credible forecasts of COVID-19 futures
Source: PLoS One. 2022 Nov 15;17(11):e0277521. doi: 10.1371/journal.pone.0277521 (PMC9665358; doi:10.1371/journal.pone.0277521)
Supplement: Attachment — Submitted filename: RefPLOS-ONE-Newcomb_Bilal_Michael.pdf [file pone.0277521.s006.pdf]

Report on

**Combining predictive models with future change scenarios can produce credible forecasts of Covid-19 futures**

By K. Newcomb, S. Bilal and E. Michael

Submitted to PLOS ONE

As the title anounces it, the authors claim that they are able to produce credible forecasts of COVID-19 futures. My opinion is that this is not the case, and the present paper does not add really useful material to the great mass of publications on this pandemic.

Of course, any reasonable model can be adjusted to past data, and varying several parameters allows to produce a variety of future predictions. That is essentially what is done in this paper, and it is far from providing realistic predictions. The statement that if immunity is permanent, there is a high probability of extinction of the pandemic in a new future, while in the case of waning immunity, it will continue for a long time, with a succession of waves, does not look as a very innovative prediction.

Two major problems about the future predictions concerning the pandemic are the loss of immunity, and the immune evasion by new variants. However, the second point is not seriously take into account, while the question whether or not some social distancing measures should be maintained, and whether or not the rate of vaccination should be multiplied by a factor 1.5 are presented as major options for controlling the future of the pandemic. These are not really serious options, since vaccination cannot be maintained at a high constant rate over a very long period of time, and most of the social distancing measures cannot be applied for ever.

Concerning the loss of immunity, the paper is very inconsistent from one section to another. In the middle of the second page of the final « Discussion » section, the authors recognize that « There is .. growing evidence .. that the effectiveness (and duration) of immunity from vaccination may differ from that induced by natural infection ». However, while it is written, 3 lines above table 1, that « the efficacy..of two-dose of mRNA vaccines.. decays to 67-80% after 7 months », the difference between « natural » and vaccine acquired immunity does not seem to be really taken into account. This 67-80 % is I think rather optimistic. What about after 10 or 12 months ? Moreover, many sections of the paper discuss the future of the epidemic in the hypothesis of permanent immunity, while others claim that a

good estimate of the duration of immunity (without differentiating between natural and vaccine acquired immunities) is 2.5 years, an estimate which I think is extremely optimistic and irrealistic.
Table 1 presents vaccine efficacies used in the simulations. But those figures are absent from the discussion in the paper.

One subsection title at the end of the « Methods » section is far from what the paper does : « Fade out probability calculations » (what is done has nothing to do with any serious probability estimation/calculation). The next section « Estimation of Population Immunity.. » contains the following assertion : « This allowed us to estimate the change of levels of immunity due to natural infection versus arising from vaccination ». I do not see which serious procedure achieves this goal in the paper.

One serious drawback is the fact that, concerning new variants, the authors insist upon their date of emergence and their transmissibility, forgetting completely their immunity evasion, which seems to be a crucial point of Omicron for example.

One problem with this paper is that it seems to hesitate between drawing conclusions from the knowledge about the pandemic as it was towards the end of 2021, and inclusion of what has been learned since.

The paper contains twice the expression « exchangeable information », the second time at the end of the discussion section, where David Aldous' St Flour Lecture Notes on Exchangeability is quoted. I do not understand what the authors mean by that expression, and how it might connect to Aldous' lectures.

For all these reasons, I recommend rejection of this paper.
