# Supplementary material for: Combining predictive models with future change scenarios can produce credible forecasts of COVID-19 futures
Source: PLoS One. 2022 Nov 15;17(11):e0277521. doi: 10.1371/journal.pone.0277521 (PMC9665358; doi:10.1371/journal.pone.0277521)
Supplement: Attachment — Submitted filename: ResponseToComments.docx [file pone.0277521.s007.docx]

**Responses to Reviewers**

**The authors thank all three reviewers for their useful comments. Below is our point-by-point response to each reviewer.**

Reviewer #1:

The paper raises an important topic: can mechanistic SEIR fitted to data be used for long-term predictions of outbreak patterns/outcomes? The big confounding factor for Covid prediction is viral evolution shaped by complex interplay between within-host biology and environmental/behavioral factors, which brings novel stains with different epidemiological profiles.

Such evolutionary forecast is beyond current understanding and modeling tools. So the paper attempts a partial answer, by combining SEIR with putative future scenarios and timely inputs on ‘novel’ strain infectivity/transmissibility profiles. The model employs case–data from Florida, and highlights multiple SEIR compartments with focus on vaccination status and schedules, and the follow-up pathways.

Large part analysis is retrospective; it involves the outbreak data through Sep 2021 (delta-variant) to simulate predicted outcomes under different vaccine/prevention scenarios (Fig. 2-8). Its primary use is demonstrating the methodology. The more relevant part includes analysis of the latest omicron wave, and possible future developments (Fig 9).
While overall exposition is satisfactory, some parts need further elaboration and additional details.

Comments:
Comment 1. General question on long-term prediction simulations. The model fitting procedure (to my understanding) involves ‘dynamic parameters’ aggregated in 10-day blocks, and weekly averaged data, in particular variable transmission rates. The key question (needs clarification) is how such dynamic (variable) parameters are projected in the future.

**Response: Thank you for the comment. When forecasting into the future, we use the best-fitting parameter sets from the final 10-day block and hold them constant. The projections are accurate up to three weeks into the future, as shown in the figure below. The model is fit to the black data, while the future data is given in red. We also run scenarios where we change the values of key driving parameters such as strength of social distancing, vaccination rates, and transmission rates, to investigate the impact of changes in these variables on the future paths of the pandemic. Such scenario analyses allow us to investigate the impact of changes in such forcing conditions.**

**Comment 2.** A related question (needs elaboration): what are the primary sources of predicted variability (uncertainty envelops shown in figs 2-8), and how they relate to calibrated parameters, social/behavioral inputs, vaccine uncertainties.

**Response: We have now added the parameter uncertainty envelopes for d and beta, the primary sources of predicted variability, in Figure S1 below. The best-fitting model ensemble consists of a wide range of these two parameters, which increases the variance of our future predictions. We do not expect vaccine uncertainties to significantly contribute to prediction error when compared to our estimates of these key variables.**

**Comment 3**. Recovered (R) compartment and the role of immunity. Based on Fig.1 hosts could lose immunity (move to VC); and immune loss rate appears in the analysis. But model equations (GitHub DE) show no loss from R (needs clarification?)

**Response: Thank you for pointing this out. We have now explicitly included the loss of immunity term and the new VS class into the model equations as shown below. Our code always contained these terms and the VS class, and we apologize for not showing this in the previous equations. We also updated the model equations on Github.**

**Comment 4.** Fig.1: multiple susceptible/vaccinated pools undergo similar SEIR- stages and transitions with asymptomatic/symptomatic pathways. Those pathways (and transitions) should depend on host’s immune status (needs more details). In addition, I’d like the authors to comment on possible effect of ‘vaccine’ and ‘natural immunity’ (R) on transmission/ disease pathways.

**Response:** **Thank you for the comment. After infections, we do assume that individuals have the same transition probabilities to constrain the number of assumptions and parameters in our model and make it tractable. However, we have incorporated the effect of vaccinations via an efficacy parameter in the transmission rates (probabilities). Because of these different vaccine efficacies, we will have a lower contribution to hospitalizations and critical cases along with death; we are therefore indirectly capturing the variation in hospitalizations, crucial cases and deaths due to vaccination in our model. Because naturally acquired immunity is more robust and lasts longer than vaccine-induced immunity in our model, we similarly also indirectly capture the difference in hospitalizations and deaths contributed by vaccine-induced vs. natural immunity in this manner. We use this formulation to approximate the differential impacts of vaccine-induced vs. natural immunity on clinical pathways because the precise details of immunity-induced transitions were not fully available at the time of writing.**

**Comment 5.** Do fig.2-3 assume full (post-infection) immunity? How different vaccinated/immune compartments (Fig. 1) combine in fig 2?

**Response:** **The reviewer is correct – in Figures 2 and 3 we assume full post-infection immunity. Therefore we do not see oscillatory behavior for the time interval shown in these figures. The predictions of cases shown in Figure 2 contain cases generated from the susceptible classes as well as all the vaccinated classes because of breakthrough infections due to imperfect vaccine efficacy.**

**Comment 6**. Elaborate uncertainty envelops of Fig.5.

**Response: When fitting the model, we generate 20,000 parameter vectors and select the 250 vectors that best fit the daily case and death data. These 250 best-fitting trajectories form the model ensemble. The median and 90% confidence interval of the predictions given by the model ensemble are given by the uncertainty envelopes shown in Figure 5. (Lines 228-234)**

**Comment 7**. Re fig 6 comments on ‘long-term oscillatory dynamics’ (repeated waves). Such patterns (convergence to endemic state via damped-oscillations) typically arise in SEIR models with immune loss.

**Response: We agree that it is well-known that damped oscillations will arise when immunity loss is applied to SEIR models. Our objective in this paper was to provide local forecasts for the state of Florida by also taking into account the impact of variations in social measures along with impacts of permanent vs. waning immunity and the advent of new variants. As can be seen in Figure 7b, these interactions can lead to some complex outcomes. For example, the size of the repeat waves caused by the new variants will be dependent on the rate of waning of immunity. A shorter waning period will result in more frequent repeat waves, and if a new variant arrives during the troughs of immunity, then larger waves could occur, as shown in Figure 7b. In addition, the waves could grow comparatively larger in size the longer out in the future the new variant arrives, and vice versa. So while repeat waves are an outcome of waning immunity, their actual sizes and timings of occurrence will be a complex outcome of interactions between the duration of immunity, and the advent and transmissibility of new variants, apart from the protective impacts of social measures.**

**Comment 8**. Omicron wave (Fig. 9) employs enhanced transmissibility (factor 1.5-2 vs. delta). Does model account for reduced cross-immunity and possible breakthrough in different vaccinated classes subjected to omicron?

**Response:** **For this work, we have incorporated a simple form of cross immunity, giving individuals immunity to future variants if they have been exposed to previous variants. We also allow for the possibility of breakthrough infections for all variants, in all vaccinated classes. It is also possible to fit different effective transmission rates for each vaccinated class, but we chose to use uniform transmission rates and differentiate vaccinated classes via an efficacy parameter to mimic breakthrough infections and reduce parameter space complexity.**

Reviewer 1 Minor comments

**Comment 9**. Multiple histories of fig 7-9 could be combined into ‘uncertainty envelops’, like in 2-6.

**Response:** **Thank you for the comment. We are showing the probabilities of elimination, which are computed by counting the individual trajectories that result in resurgence. For this reason, we chose to show all histories in Figures 5-6.**

Reviewer #2:

The manuscript "Combining predictive models with future change scenarios can produce credible forecasts of COVID-19 futures" by Newcomb analyses SARS-CoV-2 pandemic in the State of Florida. The authors develop an extended SEIR compartmental model that they calibrate to data, and then use to investigate future scenarios. Such predictions are important to anticipate the pandemic evolution, in order to timely change and adapt social measures. The authors study the impact of vaccinations, the consequences of immunity waning, and the impact of possible new variants. They conclude that new variants and loss of immunity may induce repeated waves of infection, but the severity of these waves is probably small due to a high levels of immunity acquired by vaccinations and natural infections.

In general, the study is interesting and well written. However, I have some comments that the authors might want to address in order improve the comprehension of their work, see pdf attachment

**Comment 1:** As I understand, there is no age stratification, and only one parameter for the social

interaction level. At least in the discussion the authors should comment on this, and how

this affects their conclusions, since social interactions, infections dynamics and severity of

an infection strongly depends on age.

**Response:** **Thank you for the comment. We have added text in the discussion to clarify our assumptions (Lines 747-750). We chose to neglect age stratification to restrict the number of parameters to be estimated. While our model is unable to predict age-specific outcomes, we indirectly capture the effects of age-structure through estimating the aggregate variant-specific transmission rates from the aggregate data. This allows our model to faithfully predict current and future cases and deaths at the aggregate level. We also chose to model social distancing measures by a single parameter for simplicity. While a multitude of social measures were applied in Florida at various points during the pandemic (businesses forced to temporarily close, mask mandates, work-from-home, etc.), these social measures result in a reduction in the effective contact rate between the susceptible and infectious populations. Therefore, we believe that our simple social distancing model is appropriate.**

**Comment 2:** The authors should explain in more detail how the sequential fitting is done.

Which parameters are kept fix, which are fitted, and what is the rationale be-

hind their procedure. For example, sequential fitting procedure has been implemented for France in order to control the pandemic with social measures

(<https://www.medrxiv.org/content/10.1101/2021.07.28.21260870v2>). After fixing infection parameters, only parameters for social interactions are changed for the sequential

fitting (until vaccinations and new variants emerge). However, since in this model there

seems only one parameter for social interactions, is this the parameter that has is mostly

changed ? How does their method compare to the one implemented for France ?

**Response:** **Thank you for the comment. We have clarified our sequential fitting procedure in the Methods (Lines 235-237). Our method uses a fixed calibration window of 10 days, and all parameters are allowed to adjust, including both the infection parameters as well as the social distancing parameter. The model calibrated to France’s hospitalization data (Reingruber et al.) uses an adaptive calibration window size, depending on how well the model is currently fitting the reported data. We’ve added a reference to this work in the manuscript.**

**Comment 3:** Why only daily confirmed case and mortality data is used for calibration, as stated in the

sequential model calibration section ? Why not also the daily number of hospitalisations shown in Figure 4, for example ? How it comes that the model reproduces the hospitalisation data in Figure 4a (red circles), if this data has not been used for calibration?

**Response:** **We chose to fit to confirmed cases and deaths, and not hospitalizations, in order to properly validate the model predictions. We wanted to use the hospitalization data as an independent test of model accuracy. Despite this, our model has the capacity to reproduce the confirmed hospitalization data in Florida by capturing the most important mechanisms/transition rates driving the COVID-19 pandemic.**

**Comment 4:** Figure 2A: The authors should describe in the caption what are solid and dashed lines.

Since panel (a) is about calibration, I suggest to stop the timeline at September 24 2021,

since predictions are shown in (b).

**Response:** **Thank you for the comment – we’ve revised Figure 2A.**

**Comment 5:** Figure 2b: The color coding should be as in (A), thus, the black solid line should be data.

- The color coding is confusing, I do not see yellow and blue shadings.

- There is no need to start the timeline already in Oct 2020, since it is the same in (a).

- The y axis should be restricted as in (a), there is no information beyond that range. The

peak of the blue curve can be given in the caption.

- What is the purpose of adding the case number 22400 for August 26 to the panel ? Does

it convey important information ? If yes, then it should be explained in the caption. If

not, then it should be removed.

- In the text, the figure is mentioned as Fig. S2b, which should be corrected

**Response:** **Thank you for the comment. We’ve modified Figure 2 to include these suggestions.**

**Comment 6**: Figure 3: I suggest to add Fig 3 to Fig 2 as panels (c) and (d), to gather all relevant

information in one figure 1

- Is there data than can be compared to the estimation of 30-35% for the immune population in March 2021 ?

**Response:** **Thanks for the suggestions - we have now combined these two figures. One study estimates that 66.6% of Florida’s population had natural or vaccine-induced immunity on August 26^th^ 2021 (**[**https://academic.oup.com/ofid/article/9/2/ofab647/6509923**](https://academic.oup.com/ofid/article/9/2/ofab647/6509923)**). On that date, our model estimates that 75-79% of the population has immunity, depending on the waning period (Figure 4). However, it should be noted that their estimate is not based on a mechanistic model, like ours.**

**Comment 7:** Figure 4: The figure is confusing, there are many solid and dashed curves, the yellow and blue bands look brown and violet.

- Why all these peaks in the violet band ? Why is the violet band so large ?

**Response:** **Thank you for the comment. We are selecting 250 best-fitting models to form the model ensemble, and some of these models show future peaks, especially when social measures are released. To illustrate, we’ve included all 250 models in the figure below to show the variance in predictions. The band is large due to uncertainty in our knowledge of the model parameters.**

- Is there no hospitalisation and death data before June 2021 ?

**We are highlighting the close matching of the hospitalization and death data to our model predictions during the Omicron wave. We use the entire death data from March 2020 to September 2021 to fit the model, while the hospitalization data is left out for validation.**

- What is the point of highlighting these numbers for August 30 September 8 in the two panels

**We’ve removed the text from Figure 4, as suggested by the reviewer.**

**Comment 8**: Figure 5: Are the simulations the same as in Fig 2, or are they refitted to the data for

each waning period ?

**Response: The simulations are not the same – they are refitted to the data for each waning period.**

- How are the waning numbers calculated? With an exponential distribution with time

constants 1, 2.5 and 5 years?

**Response: The waning rate is used to compute the flow of individuals from the recovered classes back to the susceptible state (dR/dt = -(waning_rate)*R). This results in an exponential decay of recovereds.**

- In Fig 2 without waning, the release of socials measures did not lead to a pan-

demic increase. Why such a strong reaction now with waning, what generates the

instability after September 24 in presence of waning ? Why are the peaks occurring so

shortly after September 24 although the waning periods are much larger ? The brief

explanation given in the text is not sufficient for a comprehension.

**Response: We refit all model parameters when waning of immunity is assumed. This results in different parameters being selected during the fitting process. In particular, the model estimates of social measures are higher when waning of immunity is assumed. Therefore, when social measures are released, the large pool of susceptibles (due to waning) leads to a much larger wave.**

- It would be more comprehensive if the color coding in this figure would be as in Fig 5,

thus, black and blue colors distinguish the two scenarios.

**Response: Figure 5 follows the same color scheme of Figure 2 – the black curve represents the median model prediction given current social measures, while the blue curve represents full release of social measures.**

**Comment 9:** I suggest to merge Fig 6 with Fig 5

**Response: Thank you - we have now merged Figures 5 and 6.**

**Comment 10:** "We calculated and used the RMSE values of fits of our models to the case data observed

over a 4-week period around the peak of the 4th (delta) wave to detect signals for the

emergence and operation of waning immunity." To this reviewer it is unclear what exactly

has been done.

**Response: Thanks for the comment. When a 2.5 year waning period was applied, the fit resulted in the lowest RMSE. We are noting that this suggests a 2.5 year waning period. We’ve changed the language in the text. (Lines 445-446)**

**Comment 11:** Figure 7: It is unclear how all the different curves are obtained. Which parameters are

varied, and how ? Before September 2021, all the different scenario reproduce the data.

After September 2021, they start to diverge. Does this mean, that the previous data

was not sufficient to constrain all the model parameters ? Which parameters are most

important for the divergence observed after September 2021 ? Which additional data

would be helpful to constrain these parameters to reduce the prediction divergence?

**Response:** **The parameters most responsible for the model variance are the transmission rates for each variant, and the social distancing strength parameter. We use a wide range of priors to allow the model to fit a wide range of transmission conditions, and this reflects our uncertainty in the true values of these parameters. The reviewer is correct when stating that this results in a divergence of predictions across parameter sets. We have revised Figure S1 (see response to reviewer #1, comment #2) to show the confidence interval and median predictions of the average transmission rate and social protection factor.**

**Comment 12:** Figure 8: Same as Figure 7. Which parameter uncertainties or fluctuations causes the

large divergence seen after September ?

**Response:** **As we mentioned in the response to comment #11, the main parameter uncertainties are in the transmission rates and the social distancing parameter (see Figure S1 in response to reviewer #1, comment #2).**

**Comment 13:** Section "Impact of new variants": I did not understand what exactly the parameter

variance maintenance mechanism does, and how it affects the model.

**Response: Our sequential fitting algorithm uses the posteriors from the previous 10 days to form the priors for the following 10 days. This results in a narrowing of the set of parameters that the model has access to. Without adding draws of parameters from the original prior ranges, the algorithm will tend to lose the ability to fit to rapidly changing transmission conditions.**

**Comment 14:** Figure 9a: Data is now available until February 2022. Why has the model not been

calibrated to reproduce the whole data, since the simulation from September until January does not agree well with the data. Why ? In principle, the model should be calibrated to accurately reproduce all available data, before being applied and modified to make predictions. Such a procedure that continuously calibrates the model to available data in order to make the best predictions has been developed for France (<https://www.medrxiv.org/content/10.1101/2021.07.28.21260870v2>).

**Response:** **For the purpose of this publication, we deliberately chose to fit the model to September 2021 in order to see how well the model could capture the Omicron wave, if the transmission rate and date of emergence are known in advance. We agree with the reviewer – when the most up-to-date forecasts are required, we apply the latest dataset to train the model, as we do when we inform policy making.**

**Comment 15:** "Given that the best prediction was accorded by the November 1st emergence model, the data also suggests that a lead time of at least 6 weeks is required for the provision of

such information to allow the reliable making of such a prediction." Is 6 weeks something

general, or specific to this model? What are the implications of this for reliable predictions

? We need past data from at least 6 weeks?

**Response:** **We ran many simulations of the Omicron variant in which we varied the date of emergence. We were able to mimic the Omicron wave by seeding the variant six weeks prior to the peak. This suggests that early estimates of the transmissibility of a new variant could help inform predictive models, which could in turn forecast the peak cases, hospitalizations, and deaths caused by the impending wave. The 6 weeks figure is not a general principle, but is specific to our model and COVID-19 transmission in Florida.**

**Comment 16:** The Discussion is quite lengthy. I suggest to make it more crisp and to better highlight

the main findings.

**Response:** **Thank you for the comment. We’ve modified the discussion to better highlight the main findings.**

Reviewer #3:

As the title announces it, the authors claim that they are able to produce credible forecasts of COVID-19 futures. My opinion is that this is not the case, and the present paper does not add really useful material to the great mass of publications on this pandemic.

**Response: At the time of writing, it was unclear how the future path of COVID-19 pandemic will play out. This was because of the uncertainty regarding the combined effects of variations in social measures, levels and duration of immunity, and new variants will impact the likely future paths of the pandemic. This paper, in our mind, is the first to address precisely the interactions between these effects on future pandemic outcomes. At that time, there was thought that models fit to previous data would not be able to forecast the future reliably. We show in this paper that provided we meet certain conditions (in particular, guarding against sample depletion and timely provision of data with a reasonable lead time, see below), models such as ours do contain information that would be useful to predict how the future will look for COVID-19. This is a valuable insight and surely of relevance to using models such as ours for modeling the future of pandemics. For these reasons, we do not agree with the author’s opinion that the present paper does not add useful material regarding modeling the pandemic.**

Of course, any reasonable model can be adjusted to past data, and varying several parameters allows to produce a variety of future predictions. That is essentially what is done in this paper, and it is far from providing realistic predictions. The statement that if immunity is permanent, there is a high probability of extinction of the pandemic in a new future, while in the case of waning immunity, it will continue for a long time, with a succession of waves, does not look as a very innovative prediction.

**Response: We’ve already explored and showed that contrary to a simple SEIR model, there will be a tradeoff in the pandemic outcomes arising from the relationship between immunity and the impact of social measures (https://arxiv.org/abs/2103.06120). This shows that while with waning of immunity, we could expect oscillatory behavior in the future and not a simple fadeout, the actual future dynamics will depend on the history, current, and future changes in social measures vis a vis changes in levels of immunity. Our data-driven model allows us to capture the impact of these interactions on the future of the pandemic, in the case of Florida. In this case, therefore, the probability of extinction depends not just on the nature of immunity, but also the levels of social measures followed in a population as well as the advent and transmissibility of new variants. We’ve now added text to this effect in the manuscript (Lines 481-505).**

Two major problems about the future predictions concerning the pandemic are the loss of immunity, and the immune evasion by new variants. However, the second point is not seriously take into account, while the question whether or not some social distancing measures should be maintained, and whether or not the rate of vaccination should be multiplied by a factor 1.5 are presented as major options for controlling the future of the pandemic. These are not really serious options, since vaccination cannot be maintained at a high constant rate over a very long period of time, and most of the social distancing measures cannot be applied forever.

**Response: We do model the immune evasion of new variants via the vaccine efficacy parameter. In Figure 9, we have modeled scenarios of the arrival of new variants with increased transmissibility (1.5x and 2x the transmission rate of Omicron) to forecast what we could expect in the future with the advent of new variants. Without a credible model of arrival times of new variants, the best we could do is apply plausible scenarios as discussed. With regards to the point made by the reviewer regarding increasing vaccination rates, all we are showing in Figure 2 is that if immunity is permanent, whether we increase the vaccination rate by 1.5x or not, or release all social measures, the pandemic will simply fade-out. We are not advocating for ongoing vaccination, but merely illustrating the outcomes we can expect if immunity is permanent.**

Concerning the loss of immunity, the paper is very inconsistent from one section to another. In the middle of the second page of the final « Discussion » section, the authors recognize that « There is .. growing evidence .. that the effectiveness (and duration) of immunity from vaccination may differ from that induced by natural infection ». However, while it is written, 3 lines above table 1, that « the efficacy..of two-dose of mRNA vaccines.. decays to 67-80% after 7 months », the difference between « natural » and vaccine acquired immunity does not seem to be really taken into account. This 67-80 % is I think rather optimistic. What about after 10 or 12 months ? Moreover, many sections of the paper discuss the future of the epidemic in the hypothesis of permanent immunity, while others claim that a good estimate of the duration of immunity (without differentiating between natural and vaccine acquired immunities) is 2.5 years, an estimate which I think is extremely optimistic and irrealistic.
Table 1 presents vaccine efficacies used in the simulations. But those figures are absent from the discussion in the paper.

**Response: The reviewer must understand that we used the information regarding duration of immunity which was available at the time of writing this paper. We agree that the range of vaccine efficacies, and rates of decay of vaccine-induced immunity, could be wider than our assumptions. We fit the model with no immunity decay, and with 1 year, 2.5 year, and 5 year natural immunity decay rates. The 2.5 year decay produced the best fit to the data for natural immunity, and so we used this value in the simulations. Note that this by using this rate, immunity waning follows an exponential decay, with a time constant of 2.5 years, that is, over 2.5 years, ~63% of the recovered class will lose immunity, and over 5 years, ~86% of the recovered class will lose immunity, and so on. Therefore, we believe this value is not overly optimistic, and is consistent with new data regarding longevity of natural vs. vaccine-induced immunity for COVID-19. Regarding vaccine-induced immunity, at the time of writing, we used the best information available to us. However, the latest empirical information does support that the efficacy of vaccine-induced immunity will decrease over time (https://www.nature.com/articles/s41467-022-30895-3). We have to consider this in future work, but note breakthrough infections due to lowered vaccine-induced immunity will, after a lag, translate to the generation of higher natural immunity, which is more robust and longer lasting. This would likely produce long-term forecasts that are more optimistic than shown in this paper.**

One subsection title at the end of the « Methods » section is far from what the paper does : « Fade out probability calculations » (what is done has nothing to do with any serious probability estimation/calculation). The next section « Estimation of Population Immunity.. » contains the following assertion : « This allowed us to estimate the change of levels of immunity due to natural infection versus arising from vaccination ». I do not see which serious procedure achieves this goal in the paper.

**We have explained how we calculated the probability of resurgence using simulation, which is a valid method. We do not understand what the reviewer means by “serious probability estimation”.**

One serious drawback is the fact that, concerning new variants, the authors insist upon their date of emergence and their transmissibility, forgetting completely their immunity evasion, which seems to be a crucial point of Omicron for example.

**Response:** **We model new variants using progressively higher immunity evasion. Our analysis shows that the effect of transmissibility and date of emergence have a more significant effect on case incidence as compared to immunity evasion. However, we agree with the author that that if a new variant is also more immune evasive, then it could increase the size of the future waves. The precise increase will need to be investigated in relation to the dynamics of naturally acquired vs. vaccine-induced immunity as well as their interactions with social measures to obtain a fuller description of such immune evasiveness on the future of the pandemic. We have now described this caveat in the text (Lines 714-718)**

One problem with this paper is that it seems to hesitate between drawing conclusions from the knowledge about the pandemic as it was towards the end of 2021, and inclusion of what has been learned since.

**Response: It is hard to understand what the reviewer is alluding to, in terms of hesitancy. We’ve included all relevant features of the pandemic, including the relative transmissibility of new variants, the arrival of new variants, and the state-specific vaccination rates, including multiple doses. This has allowed us to model Omicron and the future subvariants, and our simulations have been useful in informing local policy making.**

The paper contains twice the expression « exchangeable information », the second time at the end of the discussion section, where David Aldous’ St Flour Lecture Notes on Exchangeability is quoted. I do not understand what the authors mean by that expression, and how it might connect to Aldous’ lectures.

**Response: As we’ve explained clearly in the paper, in the introduction as well as discussion, exchangeable information here is meant to determine if our model fits sequentially to previous data, as both the structure and parameters, to make reliable forward forecasts. That is, whether the learned parameters from previous data will allow us to make predictions of the future. We show and discuss that our model can do so, provided that we include samples of our original parameter priors during the updating process, and we know the timing of the arrival of new variants. This shows that even in a nonergodic system, novel futures could be approximated provided the space of parameters isn’t depleted by the past, to allow information contained and captured by our model to be exchanged in order to simulate the future. It is beyond the scope of this paper to investigate this in detail, but we wanted to highlight that models such as ours can be used to project the future, provided the above conditions are met.**
